# Sol-Gel Derived Dual-Functional Organosilicone Coating for Enhanced Solar Panel Performance

**DOI:** 10.3390/gels12040316

**Published:** 2026-04-08

**Authors:** Jianping Huang, Xinyue Liu, Junjie Liu, Ling Yang, Jiang Li, Ziya Bai, Qingfei Zhao, Jinzhi Tong, Tiezheng Lv

**Affiliations:** College of Materials Science and Engineering, Hunan Institute of Technology, Hengyang 421200, China; hjphit_1983@163.com (J.H.); lxyhnit@163.com (X.L.); jjliu2003@163.com (J.L.); 18274489028@163.com (L.Y.); 15080822781@163.com (J.L.); 19892552660@163.com (Z.B.); 18531011246@163.com (Q.Z.); 19971878910@163.com (J.T.)

**Keywords:** glass, non-typical luminescence, anti-reflection, organosilicone, solar panel efficiency

## Abstract

In this study, a non-typical luminescent organosilicone was synthesized through a click reaction and used as a cross-linker to cure hydroxyl-terminated dimethylsilicone oil at room temperature via the sol–gel process, followed by application as a coating on a glass surface. This organosilicone film functions effectively as a luminescent down-shifting (LDS) material. Additionally, the presence of methyl groups and voids in the structure imparts a low refractive index, allowing it to serve as an anti-reflective (AR) layer. Optical and structural analyses on organosilicone-coated glass samples were conducted, and the dual-functional layer was applied to the glass cover of a perovskite solar panel to evaluate its performance. The coating not only enhanced light transmission as an AR layer but also converted UV light into blue light, which was absorbed by the solar cell. The results indicated improved solar panel performance, particularly in short-circuit current (Isc), external quantum efficiency (EQE) in the UV wavelength range, and overall efficiency. Overall, this material is a promising candidate for solar panel applications owing to maximized UV absorption for LDS, preserved transparency of the top cover glass, and room-temperature gelation, which facilitates repair of the dual-functional coating.

## 1. Introduction

Solar cells are photovoltaic (PV) devices that absorb sunlight and convert it into electricity. Whether based on crystalline silicon (c-Si) or thin-film materials like perovskite [1,2,3] or copper indium gallium selenide (CIGS), these cells are vulnerable to harsh outdoor environments. To protect them, they are typically encapsulated in transparent glass and sticky transparent polymer to form solar panels for long-term application. Glass, as a rigid protective layer, offers several advantages for solar panels, including humidity and salt-mist blocking as well as corrosion resistance. Despite these benefits, PV glass introduces several potential optical losses, primarily due to interface reflection [4], surface contamination [5], and intrinsic absorption. Minimizing these optical losses is a cost-effective and straightforward approach to enhancing the power output of solar panels [6]. Consequently, the application of a porous silica-based anti-reflection (AR) coating, often applied via sol–gel chemistry, has become standard in solar panel manufacturing [7]. Float glass produced through high-temperature manufacturing processes is commonly used in the PV industry. PV glass is typically composed of SiO_2_, Na_2_O, and Al_2_O_3_, which induce high transparency. However, these components do not maintain high transparency across the entire solar spectrum (250–2500 nm), especially in the ultraviolet (UV) range below 400 nm, where transmission decreases significantly as the wavelength shortens [8]. This results in an optical loss of approximately 4–6% across the solar spectrum in the UV range due to the glass covering. Modifying the glass composition to address this issue is impractical for mass production. Photons with wavelengths between 250–400 nm interact strongly with the front layers of PV devices [9,10], contributing to light-to-current conversion. In addition to generating electron-hole pairs, the excess energy of UV photons is converted into heat through phonon generation, which raises the operating temperature of the solar panel. This temperature increase accelerates the degradation of Ethylene-Vinyl Acetate Copolymer (EVA) and solar cells, thereby shortening the lifespan of solar panels, which are typically warranted for a minimum of 25 years. To mitigate this issue, UV absorbers are often embedded within the EVA layer to block UV light. As a result, the functional wavelength range for most solar cells begins at around 400 nm. Overall, UV light from solar radiation is not efficiently utilized: most of it is blocked by the glass, while the portion that does penetrate contributes to thermal aging issues. Thus, there is a pressing need to find effective solutions to address these UV-related challenges. Luminescent down-shifting (LDS) technology, which has been explored for several decades, offers a promising approach. LDS converts unusable UV light into longer-wavelength visible light, enhancing the efficiency of photovoltaic devices.

Various LDS materials have been studied and evaluated [11,12], including semiconductor silicon quantum dots [13], carbon quantum dots (CQDs) [14], and organic dyes [15]. These materials have demonstrated enhanced cell efficiencies due to the down-shifting of incident light. However, none of these materials is ideal for LDS applications in PV glass. Due to their intrinsically colored functional groups, they are not fully transparent, even at low concentrations, which typically results in a reduction in glass transmittance. Additionally, these LDS materials often lack stability under outdoor conditions; exposure to harsh weather, such as rain, sandstorms, or wind, can degrade them over time. To extend the lifetime of the LDS effect, these luminescent materials are commonly encapsulated within the EVA layer beneath the glass cover. However, because the glass already blocks most UV radiation, the LDS effect in this configuration is minimal. For LDS materials to be effectively integrated into standard solar panel processes and fully utilized, three key requirements must be met: (1) the materials must be transparent, without compromising transmission; (2) they should be located on the outer surface of the PV glass to maximize UV absorption; and (3) the LDS layer should be easily repairable to ensure long-term functionality. Thus far, most LDS research has focused on solar cells [16] and neglected solar panels that use glass as a protective top cover. The issue of reduced glass transmittance due to the incorporation of LDS materials has been recognized, as has the problem of refractive index mismatch. In PV glass applications, developing materials with a low refractive index is critical. The prevailing silica sol–gel process achieves a refractive index as low as 1.38 to 1.39 [17]; however, most LDS materials have a refractive index higher than this range. When LDS material with a porous silica layer as a multilayer structure is applied to PV glass, it inevitably compromises the AR properties. Consequently, the optical gain from the LDS effect cannot compensate for the reduction in AR performance. Therefore, achieving dual functionality combining both AR and LDS effects on the glass surface presents an intriguing area for future solar panel development.

Organosilicone is a novel member of the silica family, featuring a hybrid structure with repeating Si-O units in the main chain and organic functional groups attached to the silicon atoms as side chains. This unique structure enables strong adhesion to silica surfaces, while the organic side chains impart desirable properties, such as hydrophobicity and transparency. Unlike traditional silica gels derived from Tetraethyl orthosilicate (TEOS), which form brittle inorganic frameworks, the covalent bonding of methyl and mercaptopropyl groups within the Si−O−Si backbone modifies the gel’s physical properties. The organic groups act as internal spacers, preventing the dense packing of the silica network. This “organic modification” is what grants the gel its flexibility, strong adhesion to the glass, and resistance to cracking, essential properties for outdoor applications on solar panels. Due to these distinctive characteristics, silicone has found applications in various fields. For instance, previous research has demonstrated that a porous methylsiloxane layer, formed through a dealcoholization cross-linking reaction, can function effectively as an AR layer for PV devices [18]. Additionally, silicone is widely used as a mature encapsulation material for frame sealing [19]. Organosilicone with non-traditional luminescent properties has also been extensively studied and evaluated as a fluorescent source in biological research [20]. These non-traditional luminescent silicones are typically synthesized via thiol-ene click chemistry, a simple and efficient method for formulating various alkoxysilanes [21,22]. This reaction preserves the silicone’s high transparency since it does not require the addition of colored luminophores while introducing luminescent properties, although the luminescence spectra and efficiency remain suboptimal. Conventional luminescent materials, such as CdTe quantum dots or organic dyes, are often embedded within solid matrices like epoxy or silica, where they are prone to aggregation, leading to luminescence quenching. Although luminescent materials have been extensively investigated for various types of solar cells, their integration into standard solar panel manufacturing has rarely been explored due to conflicts with glass transparency. Organosilicone, with its non-traditional luminescence properties, offers several advantages for PV applications, including resistance to luminescence quenching, environmental sustainability, low fabrication costs, intrinsic transparency, robust thermal stability, and strong adhesion to the silica glass commonly used in solar panels.

Using this simple and effective approach, we fabricated a functional alkoxysilane via UV irradiation-induced thiol-ene click chemistry as a cross-linker. This alkoxysilane was employed for room-temperature gelation of hydroxyl-terminated dimethylsilicone oil to form a solid optical AR thin film with a low refractive index. Furthermore, the cross-linker imparts non-traditional luminescent properties, introducing a new LDS function without reducing transmittance. A comprehensive set of characterization experiments, including morphological, structural, and optical property measurements, was conducted. Notably, if the LDS layer on the glass surface is damaged, it can be re-coated as a repair solution, thanks to the room-temperature curing capability. This recoatability significantly enhances the weather resistance of the functional organosilicone layer, making it well-suited for PV applications. We obtained the synergy effects of AR and LDS using a modified alkoxysilane in the organosilicone coating on PV glass. Its room-temperature curable property also extended its application range.

## 2. Results and Discussion

### 2.1. Material Characterization

**Figure 1:**
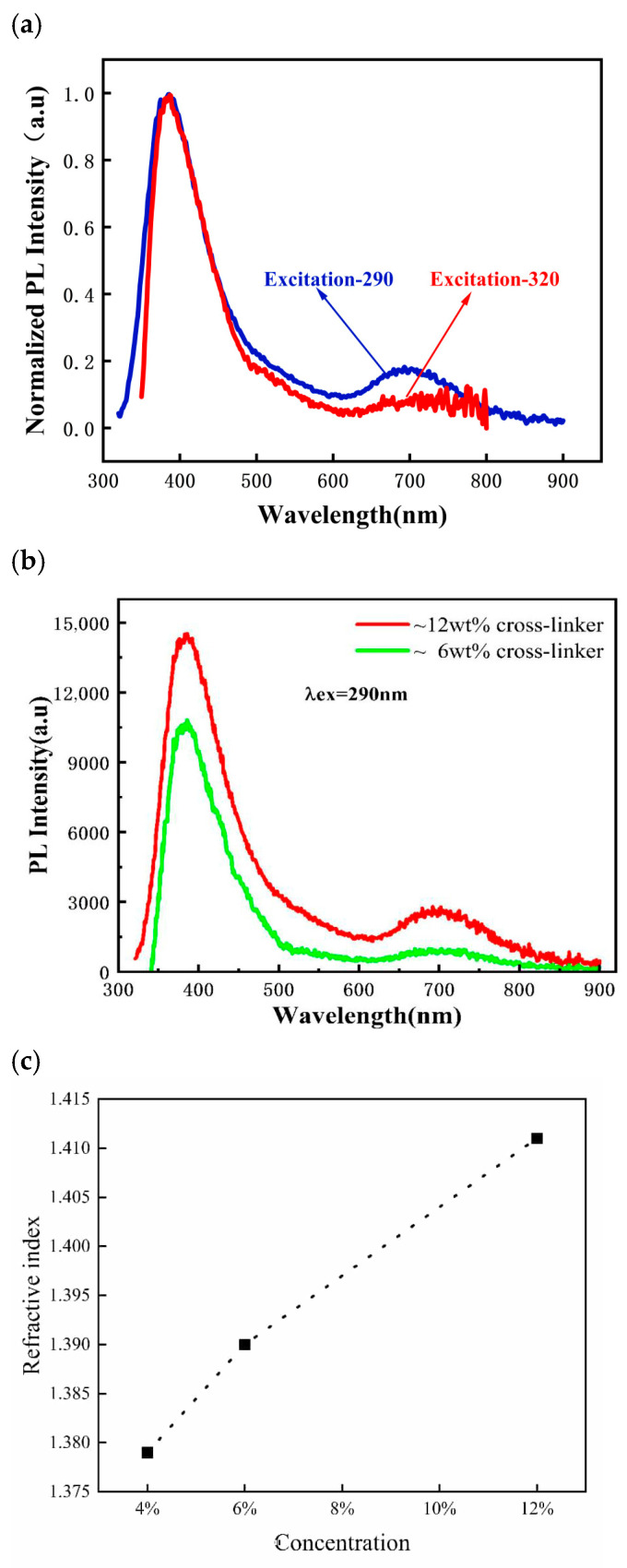
(**a**) PL spectra excited by different 290 and 320 nm, respectively; (**b**) PL spectra excited by 290 nm in conditions of 6 and 12 wt% cross-linker, respectively, and these down-shifted luminescence emission peaks centered around 420 nm; (**c**) refractive index plot as a function of concentration of cross-linker.

All optically tested samples were composites of dual-functional organosilicone coatings on glass substrates. The luminescent properties were evaluated by recording photoluminescence (PL) spectra using an Edinburgh FLS1000 fluorescence spectrometer, EI, Edinburgh, UK, over a wavelength range of 300–900 nm, with excitation sources at 290 and 320 nm. Absolute photoluminescence quantum yield (PLQY) measurements were obtained using an integrating sphere connected to an Edinburgh FLSP920 instrument, EI, Edinburgh, UK. Quinine bisulfate (PLQY = 54% in 0.1 M H_2_SO_4_) served as the reference. The measured PLQY of the sample containing 7% cross-linker was 15.68%. Each sample point was measured five times, and the averaged results are presented in Figure 1.

Organosilicone materials have a range of optical applications due to their versatile optical properties. The microstructure of the sol–gel-derived organosilica film plays a pivotal role in minimizing optical losses; for instance, a transparent gel indicates that the network is homogeneous at a scale much smaller than the wavelength of visible light, and the rigid matrix of the gel “locks” the cross-linker molecules in place, reducing non-radiative decay pathways and stabilizing the luminescent centers. Organosilicones with high-refractive-index phenyl groups are commonly used as encapsulation layers for LED chips [23], while hydrophilic polyurethanes modified for high oxygen permeability are used in contact lenses [24]. However, applications utilizing organosilicone’s luminescent properties are limited to only a few fields. One example involves the use of thiol-ene “click” chemistry to create imidazole-functionalized polysiloxanes for Fe^3+^ detection through luminescence quenching [25]. Extending the luminescent properties of organosilicones, such as intensity, lifetime, and spectral matching, requires further investigation [26]. In this study, the synthesized organosilicone achieved a PL quantum yield of 15.68%, which is lower than that of previously reported LDS materials [27]. This suggests potential for improvement, possibly through alternative reactions such as thiol-yne click chemistry in addition to the thiol-ene reaction [28]. As shown in Figure 1, the luminescence exhibits an excitation-independent behavior: whether excited at 290 or 320 nm, the emission wavelength remains constant at around 410 nm. Previous studies have investigated the origin of this non-typical luminescence in organosilicone, and this model is still under further optimization [29]. The luminescence may arise from the coordination of lone-pair electrons on sulfur atoms with silicon atoms, which modifies the five-fold degenerate 3D orbital of silicon, causing a split in energy levels. The photoluminescence results from electron transitions from these split 3d orbitals to the ground state, maintaining a constant band gap. This property enables the synthesized organosilicone to absorb UV light across a range of wavelengths and emit consistent blue light. In addition to cross-linking with dimethylsilicone oil, the synthesized luminescent organosilicone can also self-cure through the hydrolysis and subsequent condensation of methoxy (OCH_3_) groups. The presence of excess cross-linker does not interfere with the layer formation, providing flexibility in controlling UV absorption and photoluminescence. Since UV absorption depends on the concentration of the luminescent cross-linker, increasing the cross-linker concentration enhances luminescent intensity, as shown in Figure 1b. However, we measured the refractive index as a function of concentration using ellipsometry, and as shown in Figure 1c, an excessive concentration of luminescent cross-linker could increase the refractive index of the organosilicone layer, which would compromise its anti-reflective (AR) performance. Therefore, optimizing the concentration of the luminescent cross-linker is essential to balancing AR and LDS effects. The luminescence properties of the synthesized organosilicone still have room for enhancement. The emission wavelength is influenced by the splitting of silicon’s 3d orbitals, which is determined by the coordination field around the silicon atoms. In addition to mercaptopropyl groups, aminopropyl-functionalized siloxanes could also undergo “click” reactions, potentially altering the coordination environment and further splitting the 3d orbitals. This approach could shift the emission to longer wavelengths, increasing both transmission and internal quantum efficiency (IQE) compared to the current 410 nm emission. Based on the above characterizations, a 4% concentration of the luminescent cross-linker was selected for fabricating the dual-functional organosilicone layer in subsequent PV tests.

**Figure 2:**
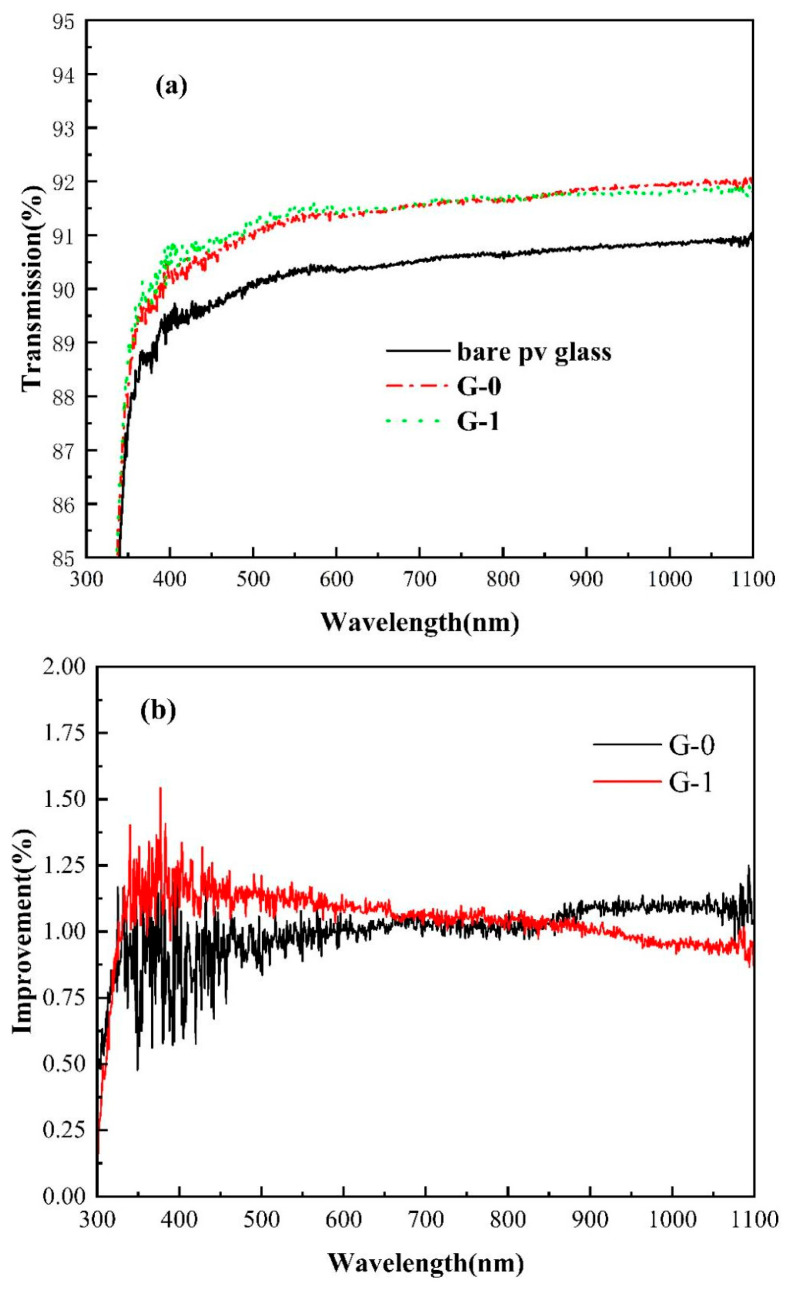
(**a**) UV-Vis transmission spectra of three different glass samples; (**b**) transmission spectra enhancement comparison for G-1 and G-0 based on bare PV glass.

UV-visible spectra of G-0 and G-1 were recorded using a UV-1601PC, Shimadzu Corporation, Kyoto, Japan, to evaluate transparency retention after applying the dual-function organosilicone coatings. The transmission and improvement curves of G-0 and G-1 are shown in Figure 2a,b, respectively.

The UV-Vis transmission spectra of G-0 and G-1 are shown in Figure 2a. As illustrated in Figure 2b, the average transmission improvement compared to bare PV glass across the entire wavelength range is approximately 1%, attributable to the lower refractive index of the coating layer. Comparing G-0 and G-1 in Figure 2a, the spectra shapes are nearly identical; however, G-1 could not keep broadband higher enhancement than G-0, as demonstrated in Figure 2b. This is likely due to other important but hardly controlled factors of the blade-coating process at the lab level, such as coating speed, slurry composition uniformity, and so on. This reduction suggests that maintaining a consistent thickness and composition of the coating will be crucial for stable performance in future mass-production applications. Previous studies have shown that different organic groups attached to the silicone main chain can influence its refractive index, with methyl and fluorine groups resulting in lower values, and phenyl or mercaptopropyl groups producing higher values. In our organosilicone formulation, less than 7% mercaptopropyl was incorporated during curing, with more than 90% of the composition comprising methyl groups. Additionally, the inherent “void” structure at the molecular level was a consequence of the gelation process. Finally, the refractive index of the formed organosilicone layer remains lower than 1.38.

**Figure 3:**
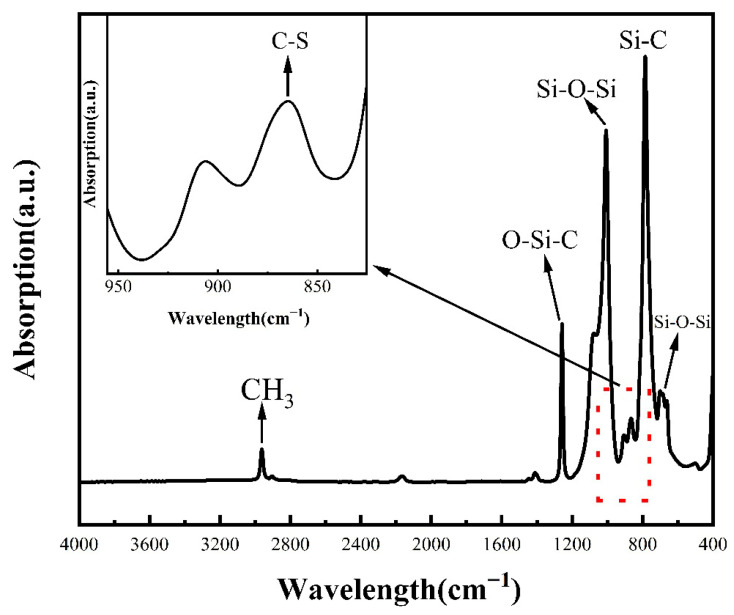
FTIR spectra for the glass with this organosilicone coating. The chemical structure peaks formed by siloxane coatings, such as C-H, Si-O-C, and C-S, are clearly visible.

Fourier-transform infrared (FTIR) spectroscopy was performed using a Thermo Scientific Nicolet iS20 spectrometer, Thermo Fisher Scientific Inc., Waltham, MA, USA in attenuated total reflection (ATR) mode to examine changes in the composition and structure of the glass surface after applying the dual-functional organosilicone coating. The FTIR results for G-1 are presented in Figure 3. The FTIR spectra display several key structural signals, including a peak at 2960 cm^−1^ attributed to the CH_3_ group, a peak at ~1260 cm^−1^ corresponding to the anti-symmetric stretching vibration of the O-Si-C bond, and a peak at ~780 cm^−1^ related to Si-C bonds. These features confirm the presence of methylsiloxane, which, as previously discussed, is essential for maintaining a low refractive index. Additionally, no peak was observed at ~810 cm^−1^, which would indicate the presence of S-H bonds; these were cleaved during the thiol-ene click reaction. However, a relatively weak peak at ~860 cm^−1^ corresponding to the C-S bond was detected, providing evidence of the luminescent properties of the coating.

**Figure 4:**
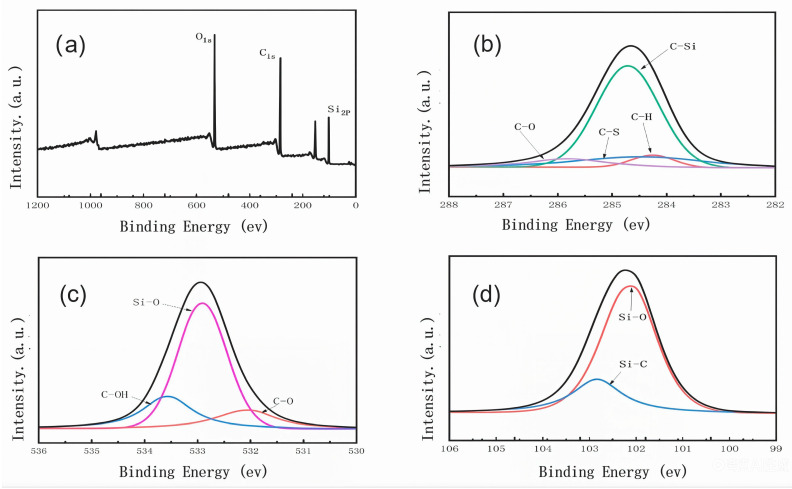
(**a**) X-ray photoelectron spectroscopy (XPS) survey scan for PV glass coated with dual-function silicone coating, (**b**) C1s spectra, (**c**) O1s spectra, (**d**) Si 2p spectra.

X-ray photoelectron spectroscopy (XPS) survey profiles of G-0 and G-1 were also obtained using a Thermo Scientific K-Alpha spectrometer to analyze the atomic composition of the methysiloxane-coated glass, as shown in Figure 4.

The XPS profiles shown in Figure 4a reveal prominent peaks for C 1s, Si 2p, and O 1s, all originating from the silicone coating on the glass. The deconvoluted C 1s spectra in Figure 4b indicate the presence of C-O (285.88 eV), C-H (284.32 eV), C-Si (284.73 eV), and C-S (284.58 eV) bonds. In the Si 2p spectra shown in Figure 4c, peaks at binding energies of 102.84 and 102.13 eV correspond to Si-C and Si-O bonds, respectively, further characterizing the siloxane structure. The O 1s spectra in Figure 4d confirm the presence of Si-O, C-OH, and C-O bonds. The C-S bonds observed in Figure 4b provide further confirmation of the thiol-ene click reaction used in the synthesis.

### 2.2. PV Performance

**Figure 5:**
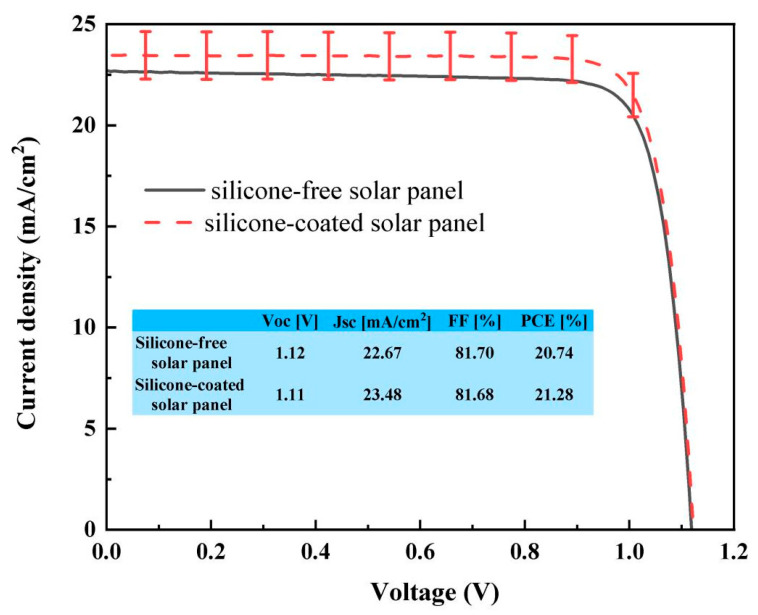
I–V characteristics of mini perovskite solar panel without (black curve) and with dual-function silicone coating (red curve). The inset table shows that there is around 0.81 mA/cm^2^ improvement in short-circuit current (Jsc) of the mini perovskite solar panel with dual-function silicone coating.

The performance enhancement of solar panels with the dual-functional organosilicone coating was assessed by simulating the encapsulation of a single perovskite solar cell (dimensions: 1.5 × 1.5 cm^2^) using standard PV glass. This glass was laminated onto the solar cell to form a mini-panel through vacuum hot pressing at 160 °C for 10 min. Current–voltage (I–V) measurements were performed using a solar simulator (IPCE 7-SCSpec Newport 94023A) under AM1.5 standard conditions. The mini-panel was measured both with and without the organosilicone coating, and the resulting I–V curves were measured to evaluate PV performance, with error bars shown in Figure 5 for comparison of the effects of the silicone coating. The PV test parameters are tabulated in the inset. Details of the solar panel fabrication process are not provided here, as they are not directly relevant. As shown in Figure 5, the dual-functional silicone-coated samples exhibited improvements in short-circuit current density (Jsc), increasing from 22.67 mA/cm^2^ to 23.48 mA/cm^2^, and in total efficiency, rising from 20.74% to 21.28%, with other parameters remaining nearly stable. These improvements are likely due to the enhanced light transmission through the standard TCO (Transparent Conductive Oxide) glass when coated with the dual-functional silicone layer. Perovskite solar panels, in particular, have a stronger response to blue light compared to crystalline silicon solar panels [30]. Additionally, for the perovskite cells, a significant efficiency increase (up to 40%) has been reported using plasmonic concentrators [31,32]. Therefore, it would be interesting to consider that this silicone layer could serve as a host for precious metal nanoparticles to achieve triple synergy. To evaluate the stability of this silicone coating, we simulated a Damp Heat (DH) test for coated glass, and samples were stored in an environmental chamber with a constant 85 °C and 85% relative humidity for 200 h [33]. In the DH test, moisture and heat usually weaken the coating quality. This result showed a notable improvement, exhibiting <2% degradation, where typical degradation is in the range of 2~5%. The DH 1000 test is normally performed for solar panels as a 25-year warranty. For the optical coating on the outer surface of glass, the typical service life is approximately 5 years; therefore, testing for 200 h is sufficient.

**Figure 6:**
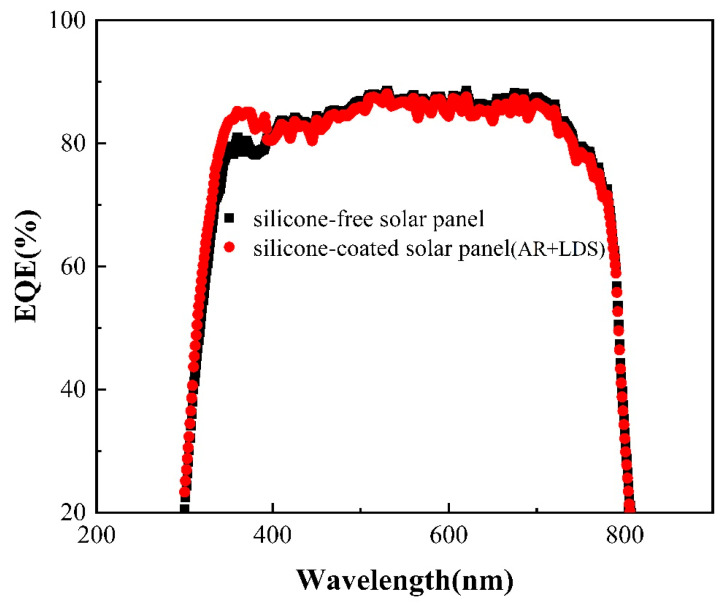
External quantum efficiency (EQE) spectra of mini perovskite solar panel without (black curve) and with dual-function silicone coating (red curve), showing that the EQE of the solar panel with silicone coating is increased in the 300–400 nm UV region.

Finally, the external quantum efficiency (EQE) curves shown in Figure 6 were measured using an Oriel Quantum Efficiency Measurement kit (QE-PV-SI) with a spot size of approximately 2 mm^2^.

The LDS function of the silicone coating also increases the effective availability of UV light in the 250–400 nm range for the solar panel, as evidenced by the improvement in the EQE curves within this wavelength range in Figure 6. However, at longer wavelengths, the EQE shows little to no significant change and even a slight reduction. Currently, the PV improvement observed in the mini perovskite solar panels is not as substantial as expected, which may be due to limited experience in encapsulating perovskite solar panels. Future studies will evaluate the effects of the coating on crystalline silicon solar panels, which offer greater long-term stability and a standardized encapsulation process. Nonetheless, the observed increases in Jsc in Figure 5 and UV response in Figure 6 provide clear evidence of the coating’s beneficial effects. Further optimization of the coating process will be necessary to demonstrate these effects on larger PV products. In addition to maximizing UV absorption and maintaining high transmission, silicone curing is a chemical condensation process, and the silicone layer can be formed at room temperature. The application of this dual-function organosilicone coating enables easy repair, potentially extending the longevity of the PV device. This reparability represents an additional benefit for PV applications. Here, we list and compare our results with the results of ref. [27] in Table 1. The high-efficiency boost in our work is likely due to maximized absorption of the coating above the glass. It is worthwhile to extend this coating to multi-junction solar cells, and LDS conversion increases the current density in the top perovskite sub-cell, improving current matching, a key parameter of tandem architecture where the current balance between sub-cells determines the overall efficiency [34,35].

Based on these measurements and the results analysis, it is evident that the various organic groups incorporated into the organosilicone framework play crucial roles in both the AR and LDS functions. The relative concentrations of these groups directly influence the effectiveness of these functions, allowing for customization of the organosilicone coating to match regional variations in AM1.5 solar spectra. In particular, the higher UV content in solar radiation at high-altitude regions could be effectively managed by enhancing the LDS function in the organosilicone coating, resulting in greater overall performance improvements.

## 3. Conclusions

A transparent and luminescent siloxane was successfully synthesized using a simple chemical click reaction, and this material functions as an effective cross-linker for curing organosilicone coatings. The organosilicone coating incorporates specific organic groups to achieve a low refractive index, enabling it to serve as an AR layer on PV glass. This coating simultaneously facilitates UV conversion and maintains high transmittance, effectively enhancing the performance of PV glass. Through a series of characterizations, we confirmed these dual effects and demonstrated a power gain on perovskite solar panels. Specifically, Jsc and overall efficiency of the mini perovskite solar panel coated with this organosilicone layer improved by 0.81 mA/cm^2^ and 0.54%, respectively. This coating offers several advantages for outdoor PV applications, including reparability, easy application, and room-temperature gelation. By addressing two optical losses with a single hybrid material, this approach also mitigates the typical durability issues associated with coatings. We believe this solution is highly effective, simultaneously addressing three issues—UV utilization, reflection loss, and coating repairability—and holding potential for widespread applications.

## 4. Materials and Methods

**Figure 7:**
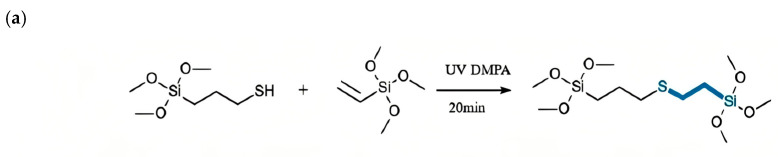

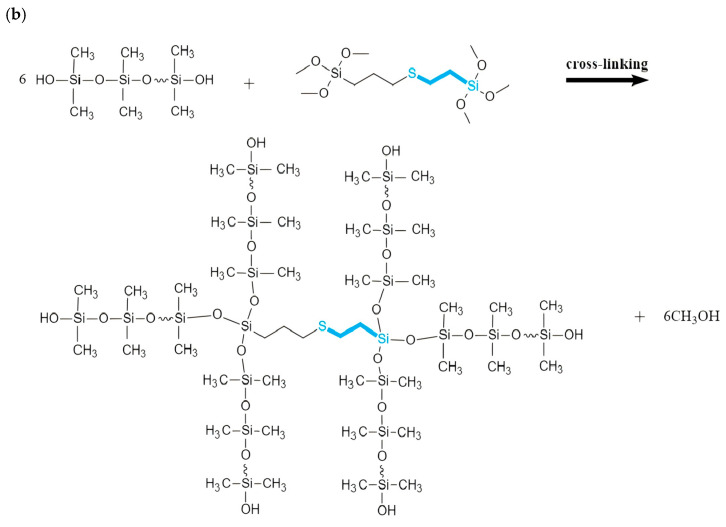
Schematic diagram for (**a**) the “click-chemistry” process between vinyltrimethoxysilane (VTMS) and mercaptopropyltrimethoxysilane (MPTMS) to form organosiicone cross-linker with blue emission; (**b**) the curing process of methyl siloxane using cross-linker formed in (**a**).

Multifunctional alkoxysilanes were prepared as outlined in Figure 7. First, the luminescent alkoxysilane cross-linker was synthesized via a photo-initiated thiol-ene click reaction. In this process, all chemicals were of analytical grade, purchased from Aladdin, Wuhan, China, and used as is. 1.47 g (10 mM) vinyltrimethoxysilane (VTMS), 1.96 g (10 mM) mercaptopropyltrimethoxysilane (MPTMS), and 1 wt% (0.0343 g) of the photoinitiator benzoin dimethyl ether (DMPA) were sequentially added to a 50 mL three-neck flask under an argon atmosphere. After thorough mixing of the reactants, the reaction mixture was irradiated with UV light for 20 min while stirring continuously to yield the multifunctional alkoxysilane. The light source was an SB-100P/F from Spectroline Corp., Melville, NY, USA with a 365 nm emission wavelength and a power intensity of 4500 μW/cm^2^. The product was a colorless, transparent, viscous liquid, the reaction process of which is shown in Figure 1a. In the second step, hydroxyl-terminated dimethylsilicone oil (optimal viscosity: 1000–3000 mPa·s) and the synthesized luminescent cross-linker were used as starting materials, with dibutyltin diacetate as the catalyst. All chemicals were of analytical grade, purchased from Aladdin, and used without further purification. The ideal weight ratio of silicone oil, cross-linker, and catalyst was 100:(3–7):1. The reaction is depicted in Figure 1b, and the coating process followed the method previously reported in Ref. [18]. After forming this organosilicone layer, which exhibits both AR and LDS functions, it was applied to commercial glass. This dual-functional organosilicone sample was designated as G-1. For comparison, a control sample using standard tetramethoxysilane (TMO) as the cross-linker was also prepared, referred to as G-0.

## Figures and Tables

**Table 1:** Summary of results of our work and comparison.

	Coating Material	PLQY (%)	Refractive Index	Position	Repairbility	PCE Boost
This paper	organosilicone	15.68	<1.38	above glass	yes	0.54%
Previous results	CQD inside silica	20.48	not listed	under glass	no	0.42%

## Data Availability

The original contributions presented in this study are included in the article. Further inquiries can be directed to the corresponding author.

## Abbreviations

The following abbreviations are used in this manuscript:

LDS	luminescent down-shifting
AR	anti-reflective
EQE	external quantum efficiency
PV	photovoltaic
CIGS	copper indium gallium selenide
UV	ultraviolet
CQDs	carbon quantum dots
EVA	ethylene vinyl acetate
VTMS	vinyltrimethoxysilane
MPTMS	mercaptopropyltrimethoxysilane
TMO	tetramethoxysilane
PL	photoluminescence
PLQY	photoluminescence quantum yield
DMPA	photoinitiator benzoin dimethyl
IQE	internal quantum efficiency
FTIR	Fourier-transform infrared
XPS	X-ray photoelectron spectroscopy
ATR	attenuated total reflection
TCO	Transparent Conductive Oxide
Jsc	short-circuit current density
